# Chronic care management in Danish general practice - a cross‒sectional study of workload and multimorbidity

**DOI:** 10.1186/1471-2296-13-52

**Published:** 2012-06-07

**Authors:** Grete Moth, Mogens Vestergaard, Peter Vedsted

**Affiliations:** 1 The Research Unit for General Practice, Aarhus University, Bartholins Alle 2, DK ‒ 8000, Aarhus C, Denmark

**Keywords:** Primary care, Chronic disease, Multimorbidity, Workload

## Abstract

**Background:**

About 30% of the Danish population has one or more chronic conditions, and general practitioners (GPs) play a key role in effective chronic care management. However, little is known about these encounters in general practice. The aim was to describe the frequency of patients with one or more chronic conditions in general practice and how these consultations were experienced by the GPs.

**Methods:**

All GPs in the Central Denmark Region were invited to register all contacts during one day in the 12‒month study period from December; 404 (46%) accepted. For each patient contact, the GPs were asked to fill in a one‒page registration form covering information on chronic disease, reason for encounter, diagnosis, number of additional psychosocial problems raised by the patient during the consultation, time consumption, experienced burden of the consultation, referral to specialized care, and whether a nurse could have substituted the GP. Patients were categorized according to the number of chronic conditions (none, one, two, three or more) and the categories compared with regard to the GP‒experienced burden of the contacts. Moreover, we examined which chronic conditions posed the the greatest challenge to the GPs.

**Results:**

Patients aged 40 years or more had a total of 8,236 contacts. Among these patients 2,849 (34.6%; 95% CI 33.6‒35.6) had one and 2,596 (31.5%; CI 30.5‒32.5) had more than one chronic disease. The time consumption and the burden of their contacts tended to rise with the number of chronic conditions. Being present in 22.9% (CI 21.6‒24.3) of all face‒to‒face contacts, hypertension was the most common chronic condition. The burden of the contacts was experienced as particularly heavy for patients with depression and dementia due to more additional psychosocial problems and the time consumption.

**Conclusion:**

General practitioners considered consultations with multimorbid patients demanding and not easily delegated to nurses. As the number of patients with chronic conditions and multimorbidity is increasing, GPs can be expected to face a heavier workload in the future.

## Background

About one third of Danish citizens have at least one chronic disease [1] and this proportion is likely to increase over the coming years due to lifestyle, demographic development and improved treatments which allow more people with chronic conditions to live longer [2,3]. About 60‒80% of current health service resources are allocated to persons with chronic conditions and spent primarily on prescription medicine and hospital admittance [1]. It is a big challenge for healthcare systems to provide high‒quality chronic care management in an ever more specialized, technology‒based and fragmented secondary healthcare system [4,5].

Mounting evidence indicates that the general practitioner (GP) plays a key role in effective chronic care management [6,7]. In Denmark, GPs often have the main responsibility for managing this group of patients [8]. One important initiative to promote and facilitate an improvement of the chronic care management is the idea of a pro‒active care, i.e. care that seeks to identify patients with chronic conditions and to enhance initiatives in relation to systematic/guideline‒based treatment, follow‒up and monitoring of such patients [7]. This may raise the demand for health services in primary care and clearly has implications for the organization and function of general practice. However, little is known about how often patients with chronic disease contact general practice and how the GPs experience these encounters.

We aimed to describe the frequency at which patients with one or more chronic conditions are seen in general practice, their degree of multimorbidity and which chronic conditions are the most commonly presented. Moreover, we aimed to examine the complexity of care and how GPs experienced the workload associated with their encounters with patients with chronic conditions.

## Methods

We invited all GPs in the Central Denmark Region (1.2 mill inhabitants) (covering approximately 20% of the Danish population) to participate in a cross‒sectional study between December 2008 and December 2009. The GPs registered all their patient contacts on one randomly assigned date during the study period. For each patient contact, the GPs were asked to fill in a one‒page registration form covering information on chronic disease, reason for encounter, diagnosis, number of additional psychosocial problems raised by the patient during the consultation, time consumption, experienced burden of the consultation, referral to specialized care, and whether a nurse could have substituted the GP. Diagnostic information was subsequently coded using the International Classification of Primary Care (ICPC) [9]. The methods of the study has previously been described in detail [10].

### Study population

We included persons aged 40 years or more. Patients were categorised into those with none, one, two, three or more chronic conditions.

### Characteristics of the contacts

We included consultations, home visits, email contacts, and telephone consultations when counting the total number of contacts; the total number of contacts was used as the denominator when calculating the frequency of contacts. In the analysis of the characteristics of the contacts, we limited the contacts to symptom‒ and disease‒related face‒to‒face contacts (excl. telephone, email contacts and consultations on prophylactic or administrative issues).

We dichotomized the number of additional psychosocial problems in the contacts (yes, no). The GPs were asked to assess the experienced burden of the contacts (1–10, 10 being the heaviest), and afterwards we categorized theses values into three groups (1‒4 = very easy/easy, 5‒6 = medium, 7‒10 = heavy/very heavy). Referral to specialist care was divided into three groups (outpatient clinics, primary care specialists or admission to hospital).

We identified the ten most frequently registered chronic conditions and calculated the proportion of contacts for each of them. In a subpopulation of contacts with patients having only one of the ten most frequent chronic conditions, we analysed how the GP weighted the chronic disease with regard to additional psychosocial problems raised during the contact, the time consumption and experienced workload of the contacts and their possible substitution by nurses.

### Analysis

Chi‒square tests were used to examine the difference between groups having no, one, two, three or more chronic conditions. An exploratory factor analysis of the 25 most frequently registered chronic conditions was performed with an orthogonal varimax rotation of the factors to identify occurring clusters of chronic conditions, expressed as eigenvalues greater than one. The isolated burden of each of the ten most common chronic conditions was compared using Chi‒square tests, and a Poisson regression was used to estimate the prevalence ratio of the experienced burden of these ten conditions adjusted for additional psychosocial problems and time consumption. Stata 11.1 was used to perform the statistical analyses.

### Ethics

The project was approved by the Danish Data Protection Agency and by the National Board of Health. The participating GPs received a fee that was partly dependent on the number of registered contacts.

## Results

A total of 404 GPs (46.4%) consented to participate in the study. The participating GPs and the non‒ participating GPs did not differ with regard to type of clinic or the distribution of listed patients in terms of age and gender. However, the participation rate was higher for men (66.0% vs. 55.5%) and GPs with less than 20 years of service (30.2% vs. 20.0%) (data not shown).

From the overall description of all contacts, it appears that the degree of multimorbidity rose consistently with rising age (Table 1). More patients having three or more chronic conditions received home visits by their GPs, and patients without a chronic condition had more email contacts to their GPs (p < 0.001). Patients without a chronic condition had fewer follow‒up contacts than patients with chronic conditions (p < 0.001) (Table 1).

**Table 1:** Characteristics of patient contacts in general practice

		**Number of chronic conditions**				
		**0 n (%)**	**1 n (%)**	**2 n (%)**	**3+ n (%)**	**p‒value**
Overall no of contacts		n = 2,791	n = 2,849	n = 1,530	n = 1,066	
Type of contact	Consultation	1,501 (53.8)	1,577 (60.7)	860 (56.2)	582 (54.6)	P < 0.001
	Telephone	1,033 (37.0)	1,085 (33.1)	550 (36.0)	379 (35.6)	
	Home visit	34 (1.2)	65 (2.1)	61 (4.0)	59 (3.5)	
	E‒mail	211 (7.6)	113 (3.4)	50 (3.3)	40 (3.8)	
	Missing	12 (0.4)	9 (0.7)	9 (0.6)	6 (0.6)	
Gender	Male	991 (35.5)	1,127 (39.5)	616 (40.3)	406 (38.1)	P < 0.072
	Female	1,653 (59.2)	1,663 (58.4)	890 (58.2)	649 (60.9)	
	Missing	147 (5.3)	59 (2.1)	24 (1.6)	11 (1.0)	
Age groups	40–49	927 (33.2)	552 (19.4)	201 (13.1)	83 (7.8)	P < 0.001
	50–59	667 (23.9)	638 (22.4)	241 (15.8)	159 (14.9)	
	60–69	539 (19.3)	682 (23.9)	419 (27.4)	249 (23.4)	
	70+	508 (18.2)	918 (32.2)	645 (42.2)	564 (52.9)	
	Missing	150 (5.4)	59 (2.1)	24 (1.6)	11 (1.0)	
New episode or follow‒up	New episode	1,226 (43.9)	922 (32.4)	454 (29.7)	337 (31.6)	P < 0.001
	Follow‒up	1,112 (39.8)	1.628 (57.1)	912 (59.6)	655 (61.4)	
	Missing	453 (16.2)	299 (10.5)	164 (10.7)	74 (6.9)	

The number of additional psychosocial problems, time consumption and the experienced burden tended to increase with the number of chronic conditions. Substitution by nurses was considered less feasible in contacts with multimorbid patients than in contacts with no chronic condition, whereas no difference was found in referral rates to specialized care between the patient groups with none, one, two, three or more chronic conditions (Table 2).

**Table 2:** Characteristics of the contacts involving patients with one, two or more chronic conditions

		**Number of chronic conditions**				
		**0 n (%)**	**1 n (%)**	**2 n (%)**	**3+ n (%)**	**p‒value**
**Characteristics of the contacts**		n = 1,229	n = 1,315	n = 731	n = 528	
Additional psychosocial problems	Yes	110 (9.0)	225 (17.1)	161 (22.0)	118 (22.4)	P < 0.001
	No	1,119 (91.0)	1,090 (82.9)	570 (78.0)	410 (77.7)	
	No inf.	0 (0.0)	0 (0,0)	0 (0,0)	0 (0,0)	
Time consumption of the consultation	<5 min.	165 (13.4)	128 (9.7)	60 (8.2)	36 (6.8)	P < 0.001
	6–15 min.	810 (65.9)	876 (66.6)	470 (64.3)	334 (63.2)	
	16–30 min.	205 (16.9)	280 (21.3)	173 (23.7)	141 (26.7)	
	>30 min.	24 (2.0)	18 (1.4)	13 (1.8)	12 (2.3)	
	No inf.	25 (2.0)	13 (1.0)	15 (2.1)	5 (1.0)	
The GP‒experienced burden of the consultation	Easy	740 (60.2)	738 (56.1)	344 (47.1)	231 (43.7)	P < 0.001
	Medium	277 (22.5)	297 (22.6)	213 (29.1)	130 (24.6)	
	Heavy	171 [9,13]	261 (19.9)	162 (22.2)	163 (30.9)	
	No inf.	41 (3.3)	19 (1.4)	12 (1.6)	4 (0.8)	
Referrals	Primary care specialist	77 (6.3)	56 (4.3)	30 (4.1)	18 (3.4)	P = 0.098
	Outpatient clinics	75 (6.1)	64 (4.9)	38 (5.2)	41 (7.8)	
	Hospital admission	15 (1.2)	14 (1.1)	19 (2.6)	8 (1.5)	
Substitution by staff possible	Yes	144 (11.7)	231 (17.6)	111 (15.2)	83 (15.7)	P = 0.001
	No	988 (80.4)	1,003 (76.3)	568 (77.7)	416 (78.8)	
	No inf.	97 (7.9)	81 (6.2)	52 (7.1)	29 (5.5)	

^1^ Consultations on prophylactic issues are excluded.

Hypertension was the most common chronic disease with a presence in 22.9% of all registered face‒to‒face contacts (Table 3). No statistically significant clustering of chronic conditions appeared from the explorative factor analysis; this indicates the absence of particular patterns combinations of chronic conditions in multimorbid patients.

**Table 3:** **Frequency of the ten most common chronic conditions in GP face‒to‒face contacts**^**1**^

**Chronic conditions**	**n (%)**
**Hypertension**	871 (22.9)
**Type2 diabetes**	315 (8.3)
**Depression**	254 (6.7)
**Lipid disorder**	175 (4.6)
**COPD**	185 (4.9)
**Isch. heart disease**	164 (4.3)
**Atrial fibrillation**	122 (3.2)
**Obesity/Overweight**	109 (2.9)
**Asthma**	95 (2.5)
**Dementia**	53 (1.4)
**All contacts**	3,803 (100)

^1^ Consultations on prophylactic issues are excluded.

The GP‒experienced burden of the contacts singled‒out depression as the heaviest disease. Contacts with patients suffering from hypertension as the only chronic disease were the least straining and the GPs most often indicated substitution by nurses for these contacts (Table 4).

**Table 4:** Characteristics of the contacts involving patients with only one of the ten most common chronic conditions (n=660)

**Chronic disease**	**No**	**Additional psychosocial problems present n (%)**	**Time consumption Minutes n (%)**		**The GP-experienced burden n (%)**		**Nurse substitution possible n (%)**
			**<16**	**>15**	**Very easy-medium**	**Heavy or very heavy**	
**Hypertension**	304	36 (11.9)	267 (9.9)	37 (12.2)	266 (87.8)	37 (12.2)	91 (32.0)
**Type2-diabetes**	72	8 (11.1)	60 (15.3)	12 (16.7)	55 (77.5)	16 (22.5)	20 (29.0)
**Depression**	84	27 (32.1)	37 (6.0)	47 (56.0)	52 (61.9)	32 (38.1)	6 (7.6)
**Lipid disorder**	27	1 (3.7)	23 (14.8)	4 (14.8)	20 (80.0)	5 (20.0)	8 (30.8)
**COPD**	50	8 (16.0)	36 (6.0)	14 (28.0)	36 (73.5)	13 (26.5)	4 (8.3)
**Isch. heart disease**	33	3 (9.1)	28 (12.1)	5 (15.2)	31 (93.9)	2 (6.1)	6 (20.0)
**Atrial fibrillation**	27	3 (11.1)	25 (14.8)	2 (7.4)	23 (88.5)	3 (11.5)	6 (24.0)
**Obesity/overweight**	19	4 (21.1)	15 (0.0)	4 (21.1)	14 (73.7)	5 (26.3)	3 (16.7)
**Asthma**	27	2 (7.4)	20 (7.4)	7 (25.9)	26 (96.3)	1 (3.7)	4 (15.4)
**Dementia**	17	0 (0.0)	10 (11.8)	7 (41.2)	11 (64.7)	6 (35.3)	2 (11.8)
**P-values**		P<0.001		P<0.001		P<0.001	P<0.001

Table 5 shows that the presentation of additional psychosocial problems and the time consumption was associated with an experience of a heavy burden, whereas no difference in experienced burden was found between the ten most common chronic conditions per se.

**Table 5:** Association between contacts involving the ten most common chronic conditions and the GP experience of the contacts as heavy or very heavy adjusted for additional psychosocial problems and time consumption (n=660)

		**GP experienced workload as heavy/very heavy**				**P-values**
		**Prevalence (N)**	**Prevalence difference (95% CI)**	**Prevalence ratio (95% CI)**	**Adj. prevalence ratio (95% CI)**	
**Chronic disease**	**Hypertension**	0.12 (37)	ref	1	1	
	**Type2-diabetes**	0.22 (16)	0.10 (-0.001-0.21)	1.85 (1.09-3-12)	1.62 (0.94-2.8)	0.085
	**Depression**	0.38 (32)	0.26 (0.15-0.37)	3.12 (2.1-4.69)	1.41 (0.93-2.13)	0.107
	**Lipid disorder**	0.20 (5)	0.08 (-0.08-0.24)	1.64 (0.71-3.79)	1.81 (0.92-3.54)	0.085
	**COPD**	0.26 (13)	0.14 (0.01-0.27)	2.17 (1.25-3.79)	1.59 (0.94-2.68)	0.084
	**Isch. heart disease**	0.06 (2)	-0.06 (-0.15-0.23)	0.5 (0.13-1.97)	0.50 (0.13-1.90)	0.308
	**Atrial fibrillation**	0.115 (3)	0.005 (-0.13-0.12)	0.95 (0.31-2.86)	1.10 (0.39-3.09)	0.852
	**Obesity/overweight**	0.26 (5)	0.14 (-0.06-0.34)	2.15 (0.96-4.85)	1.64 (0.73-3.67)	0.232
	**Asthma**	0.04 (1)	-0.08 (-0.17-0.005)	0.30 (0.04-2.13)	0.25 (0.04-1.64)	0.149
	**Dementia**	0.35 (6)	0.23 (0.001-0.46)	2.92 (1.42-5.88)	2.00 (0.98-3.94)	0.056
**Additional psychosocial problems**	**No**	0.41 (82)	ref	1	1	
	**Yes**	0.14 (38)	0.27 (0.16-0.37)	2.8 (2.07-3.88)	1.72 (1.23-2.40)	0.002
**Time consumption**	**< 16 minutes**	0.10 (51)	ref	1	1	
	**>15 minutes**	0.50 (69)	0.40 (0.32-0.49)	5.11 (3.75-7.00)	4.06 (2.80-5.89)	<0.001

^1^Adjusted for additional psychosocial problems and time consumption.

## Discussion

### Main findings

In general practice, two out of three 40+ year‒old patients presented with at least one chronic disease. Patients with chronic conditions were more likely to raise additional psychosocial problems during the consultation than those without chronic conditions, and the GPs reported that the time consumption and the burden of these contacts tended to increase with the number of chronic conditions. Particularly demanding were contacts with patients suffering from depression and dementia. Accounting for one fifth of all registered chronic conditions, hypertension was the most commonly presented chronic condition. GPs estimated that nurses could substitute them in 32% of these contacts, but in only 7% of contacts with depressive patients.

COPD patients accounted for only 5% of the total number of contacts. This may be rather few compared with the number of contacts with hypertensive patients, because even though the prevalence of COPD is lower than the prevalence of hypertension [11,12] COPD may, indeed, be a disabling condition with a strong impact on quality of life and mortality and with a potential higher need for health services. It is therefore possible that the consumption of general practice services by patients with chronic conditions to some degree is due to some patients being aware of the importance of prevention (e.g. of hypertension) whereas others are more reticent concerning presenting at their GP. Such a difference may also be due to sociodemographic factors as COPD is shown to be associated with a low socio‒economic status [13]. Moreover, Danish GPs are paid by a capitation fee as well as fee‒for‒service and the latter makes it profitable to do many consultation checks. It cannot be ruled out that this plays a role, as hypertension checks may be easy consultations, often performed by the clinical staff. Contrary to this consultations with COPD patients according to our findings are more complex, indicating that the frequency of hypertension checks is partly defined by other factors than by a genuine need for physical care delivered by the GP.

We may expect that the rise in primary sector consultations for chronic conditions will translate into a growing workload in general practice in the future. At the same time, clinical staff is increasingly involved in substituting the GPs. However, as the staff primarily eases the workload of the GPs by handling less complicated problems, the GPs’ work with patients with complicated problems can be expected to intensify.

### Strengths and weaknesses

The present study included a large number of GPs, nurses and patient encounters and we obtained information not otherwise available about the contents of these encounters and how the GPs experienced these contacts. The study included a representative share of the GPs in the region, corresponding to 13% of all GPs in Denmark. Information about the contacts was based on the GPs’ direct experience as the questionnaires were filled in immediately after the contacts and recall bias was thereby minimised.

We were able to offer a detailed characterization of these encounters as we invited the GPs to comment on several aspects of the their experience. The number of chronic conditions may be underestimated, as the registration by the GPs may have been incomplete. The extent of this possible misclassification is unknown. Moreover, we did not present the GPs with a definition of chronic condition or disease, as we aimed in this pragmatic study to picture the activities and the daily work of the GPs as they perceived it. It may be argued that for example uncomplicated hypertension and overweight are risk factors rather than chronic conditions and should as such not be included as chronic conditions as this would imply a risk of overestimating the prevalence of chronic conditions in general practice.

Encounters in general practice may be difficult to clearly mark out with a single reason for encounter, as these contacts often are characterized by a history of GP‒patients relationship, which makes the GP take psychological factors of the patient into consideration as well as the actual reason for encounter. However, as this study was based on the GPs’ stating chronic diseases, the potential uncertainty of the character of the reasons for encounter does not hamper the results.

### Comparison with the literature

We have identified no other studies exploring how GPs perceive the workload of managing patients with chronic conditions. However, several studies have demonstrated an increase in the prevalence of psychiatric disorders in primary care. In an earlier study of our data, we found that a larger share of patients gave depression as the reason for their encounter in 2009 than in a previous study conducted 16 years earlier [10]. This rise in the prevalence of depression in general practice is supported by a study from 2008 in Dutch general practice, which showed a significant increase in the prevalence of depression and anxiety disorders [14]. Likewise, an increase in the prevalence of depression from 1998 to 2008 was found in Australia [15]. Even though these studies focused on the general morbidity in general practice and not on registration of contacts, including chronic diseases, as the present study, the results are likely to show the same pattern of an increase in the prevalence of depression in primary care.

Even though severe somatic illnesses as such as COPD will continue to be a marked challenge for the GPs it is worth being aware that GPs are likely to be facing mounting pressures in their daily work in the future due to the general rise in the prevalence of depression, stress and other anxiety disorders and complaints. Moreover, patients with depression and anxiety disorders have been documented to consult their GPs more frequently about minor ailments than patients without these psychiatric disorders, which emphasizes the complexity of the needs of these patients [16]. Further research is needed to identify the needs of these patients and how best to meet them, and studies on changes in populations’ threshold for seeking help would be relevant to shed light on whether there is a growing tendency to present the GP for minor concerns compared with earlier.

## Conclusion

GPs experienced consultations with patient suffering from chronic conditions as heavier than contacts with patients without chronic disease. The contacts involving patients with psychological chronic conditions were perceived as those that carried the heaviest workload. High workloads may affect the GP’s job satisfaction and performance. In light of the general rise in the prevalence of chronic conditions and psychological problems in general practice, future efforts should be geared to strengthened education and post‒graduate training and to preparing the GPs and their clinical staff to meet the needs of this group of patients.

## Competing interests

The author’s declared that they have no competing interests.

## Authors’ contribution

GM contributed substantially to the design, to acquiring, analyzing and interpretation of data, made the first draft and following revisions of the manuscript and has given final approval of the version to be published. MV gave substantial contribution to interpretation of data and participated actively in writing and revising the manuscript critically and has given final approval of the version to be published. PV contributed substantially to the design and interpretation of data and participated actively in writing and revising the manuscript critically and has given final approval of the version to be published.

## Declaration

The study provides documentation of the workload that management of chronic diseases poses to GPs and discloses some of the characteristics of this workload.

## Funding

This work was supported by the County of Aarhus, the Central Denmark Region; and The Danish National Research Foundation for Primary Care.

## Pre-publication history

The pre-publication history for this paper can be accessed here:

http://www.biomedcentral.com/1471-2296/13/52/prepub
